# Perceived Stress in a Gender Perspective: A Survey in a Population of Unemployed Subjects of Southern Italy

**DOI:** 10.3389/fpubh.2021.640454

**Published:** 2021-04-01

**Authors:** Chiara Costa, Giusi Briguglio, Stefania Mondello, Michele Teodoro, Manuela Pollicino, Andrea Canalella, Francesca Verduci, Sebastiano Italia, Concettina Fenga

**Affiliations:** ^1^Clinical and Experimental Medicine Department, University of Messina, Messina, Italy; ^2^Occupational Medicine Section, Department of Biomedical and Dental Sciences and Morphofunctional Imaging, University of Messina, Messina, Italy; ^3^Department of Biomedical and Dental Sciences and Morphofunctional Imaging, University of Messina, Messina, Italy

**Keywords:** perceived stress, unemployment, gender, PSS, work-related stress

## Abstract

Stressful life events, are differently handled by women and men. This study evaluates gender differences in perceived stress and health status among a sample of subjects going through a transition period from unemployment to work. This cross-sectional study enrolled 395 participants, 245 men (62%) and 150 (38%) women, between 19 and 67 years, that were going to be hired for a 6-month contract. Before being employed, all participants underwent a mandatory protocol consisting in a general medical check. Stress assessment was performed by using the Perceived Stress Scale (PSS). Most of the participants (68%) showed normal to low perceived stress level. But dividing the sample by gender, out of the remaining 32% with medium to high stress level, 11% male subjects and 22.7% females reported high perceived stress values. We found mean PSS values that are overlapping with those in the general population of developed countries. This study does not suggest an association between perceived stress and health or social parameters. However, our results highlight that the female gender is associated with higher stress level, pointing out the relevance of specific and designed interventions in the context of health promotion programs, especially in order to mitigate stress in more susceptible subjects.

## Introduction

The standard definition of stressful events identifies them as situations in which the demands are likely to exceed the resources of the individual involved (1). It is clear that stress does not inevitably produce negative consequences; but also, that high stress levels, especially when prolonged or accompanied by scarce coping resources, often result to trigger the onset of emotional and mental disorders, such as depression.

The physiological responses producing adaptation to stressful life events involve the hypothalamic-pituitary-adrenal axis and the auto–nomic nervous system, as well as their complex interactions with the metabolic system and the pro- and anti-inflammatory mechanisms of the immune system. Hyperstimulation of this network may lead to alterations in health status (1, 2).

Stress is therefore associated with physical disturbance as an increased risk of cardiovascular diseases (3, 4), metabolic syndromes (5, 6) and mortality (7, 8).

The influence of occupational status on stress is well-known and a close relationship between unemployment and psychophysical health has been demonstrated. In particular, unemployment may become a psychosocial stressor with long term consequences on work ability and the overall state of health, including stress (9). Also job search is a critical moment that can become a source of stress especially for those who cannot achieve economic and psychological independence (10), affecting the global health status. Many studies have investigated perceived stress in general populations (11–16), unemployed (17) and different categories of workers (18), concluding that unemployment and high levels of perceived stress have been associated in cross-sectional studies, but the direction of causation is unknown (19).

A large body of evidence suggests that women and men respond differently to stress and this sex difference could be explained in diverse ways. On the one hand, women not only experience more stressful life events (20) but they also have different coping strategies, that are defined as cognitive and behavioral processes used by an individual to deal with stressful conditions that are judged to be difficult, inexhaustible, hostile, harmful (21). On the other hand, these differences seem to be linked to the genes present on sex chromosomes and gonadal hormones production.

Subjective tools such as questionnaires are used to measure stress levels in workers. In the last 2 decades questionnaires like the Perceived Stress Scale (PSS) have received much attention by researchers; it emerges that the perception of work-related stress -as measured by this scale in terms of unpredictability, lack of control and overload- has a significant impact on worker's quality of life; notwithstanding its characteristic to refer only to the last month, considering that stress is a continually changing state (22, 23).

Upon these premises, this study evaluates perceived stress along with health status and associated factors with a gender perspective, among a representative sample of subjects from southern Italy going through a transition period from unemployment to work.

## Materials and Methods

### Study Design and Population

In this cross-sectional study we investigated an unemployed population that would have been hired for a 6-month contract in the context of socially useful jobs. Subjects came to our observation due to a mandatory preventive protocol required by Italian Decree no. 81/2008, preceding a temporary contract by town Municipality for socially useful jobs such as street sweeping, cleaning green areas and waste bins emptying. All the workers under the mandatory protocol were invited to participate in the study. All participants underwent a general medical check (medical history, physical examination, blood tests, electrocardiogram) between June and September 2019.

Initially, a team of well-trained physicians explained study purpose to all subjects, in order to gain their trust and to obtain the informed consent of those who accepted to participate. The presence of psychiatric illnesses was an exclusion criterion, to avoid confounding effects.

This study was carried out in accordance with the Declaration of Helsinki's ethical standards. Being part of the mandatory occupational health surveillance, the study needed no formal approval by the local Ethics Committee.

### Measures

Demographic data included age, sex, education, marital status and number of children while lifestyle and health factors included smoking, body mass index (BMI) and number of comorbidities.

The original 14-item Perceived Stress Scale (PSS-14) was developed in 1983 by Cohen et al. (22) but this first version was later revised and reduced into 10-item and 4-item versions (24–26). The Italian version of the PSS-10 was used to measure to which degree life in the previous month was unpredictable, uncontrollable and overwhelming and it is based on a 5-point response scale (0 = “never,” 1 = “almost never,” 2 = “sometimes,” 3 = “fairly often,” 4 = “very often”). After reversing the scores on the four positively stated items (Items 4, 5, 7, and 8), a PSS-10 total score was obtained by summing up all items. Higher scores indicated a higher level of perceived stress. Despite the PSS-10 is not a diagnostic instrument, it provides an output distinguishing four categories depending on different cut-off values (1–10 = “under average,” 11–14 = “average,” 15–18 = “medium-high,” ≥19 = “high”).

A medical examination was conducted before questionnaire submission in the Occupational Medicine Unit at University Hospital of Messina, starting from 07:45 a.m. (last examination started at 12:30 p.m.) It included past medical history, physical examination (i.e., blood pressure, height, and weight), blood tests (i.e., glucose, blood count) and electrocardiogram (ECG).

The subjects' chronic diseases suffered by (i.e., hypertension, diabetes, cancer, cardiovascular, respiratory, autoimmune, neurological diseases, and obesity) were considered comorbidities.

The body mass index (BMI) was calculated by dividing body weight in kilograms by the square of body height in meters and used to define persons as underweight (BMI < 18.5 kg/m^2^), normal (BMI 18.5–24.99 kg/m^2^), overweight (BMI 25–29.99 kg/m^2^), or obese (BMI ≥ 30 kg/m^2^).

To evaluate blood pressure levels of each participant, a medical doctor registered a measurement from the right arm (passively supported at the reference level of the right atrium) while the subject was comfortably seated. Threshold values of both systolic (≥140 mmHg) and diastolic (≥90 mmHg) blood pressure were applied in accordance with practice guidelines of the European Society of Hypertension and European Society of Cardiology (27).

In conformity with safety and quality standards, trained physicians performed blood withdrawals from antecubital vein by using a closed vacuum-extraction tube system. Concerning the fasting blood glucose, 100 mg/dL was chosen as the upper normal threshold according to the value ranges used by the American Diabetes Association in order to identify individuals with prediabetes (28).

ECG recordings were evaluated by a well-trained physician. QT intervals were measured manually and corrected according to the Bazett formula (Q–T corrected = Q–T interval in seconds/√R–R interval in seconds). QTc intervals between 430 and 450 ms in males and between 450 and 470 ms in females were considered borderline, QTc above these values were considered as long QTc intervals (29). Long QTc intervals indicate delayed repolarization of myocardial cells (30).

### Statistical Analysis

No formal calculation of the sample size was done, as the study was designed to include all subjects who fulfilled the inclusion criteria. Baseline characteristics were summarized using standard descriptive statistics, and a descriptive analysis was done. Continuous variables were presented as mean (SD) or median (interquartile range), as appropriate. Distributions of categorical variables were presented as frequencies and percentages. Sex comparisons of quantitative continuous variables were evaluated using the Student's *t*-test. The association between each categorical variable and sex was evaluated using the chi-square or Fisher's exact test, as appropriate.

For the purposes of the analysis, PSS-10 scores were dichotomized into no/mild perceived stress (PSS-10 < 15) and moderate/severe perceived stress (PSS-10 ≥ 15). Univariate logistic regression analysis was used to evaluate the prognostic ability of the population characteristics, individually, to predict the probability of developing moderate/severe perceived stress. Crude odds ratios with 95% confidence intervals are presented. The measure of classification accuracy of the models was assessed using the area under the receiver operating characteristic curve (AUC), also known as c-statistic (C). All hypothesis tests conducted were 2-tailed and a *p* < 0.05 was considered significant. All statistical analyses were performed using the software package SAS Studio (SAS Institute, Inc., Cary, NC).

## Results

### Population Characteristics

A detailed sample description is summarized in Table 1. Out of 485 subjects invited, 396 accepted participation (response rate 81.7%) and 1 was excluded after applying exclusion criteria. Therefore, 395 subjects were included in the study; the sample consisted of 245 men (62%) and 150 (38%) women, aged 19–67 years. The overall mean age was 40.1 years, 41.7 in men and 37.5 in women.

**Table 1:** Sociodemographic characteristics, lifestyle, and health factors of study population.

	**Study population**		**M**		**F**		
	***N***	**(%)**	***N***	**(%)**	***N***	**(%)**	***p*-value**
	395		245	(62.02)	150	(37.98)	
**SOCIODEMOGRAPHIC FACTORS**							
**Age**							
Mean	40.1		41.7		37.5		**<0.001**
Range	19–67		19–67		20–61		
**Age groups**							
19–39	207	(52.4)	116	(47.4)	91	(60.7)	**<0.001**
40–59	156	(39.5)	100	(40.8)	56	(37.3)	
≥60	32	(8.1)	29	(11.8)	3	(2.0)	
**Education**							
Illiterate	7	(1.8)	4	(1.6)	3	(2.0)	0.100
Elementary school	28	(7.1)	16	(6.5)	12	8.0)	
Middle school	240	(60.8)	159	(64.9)	81	(54.0)	
High school	113	(28.6)	64	(26.1)	49	(32.7)	
University	7	(1.8)	2	(0.8)	5	(3.3)	
**Marital status**							
Unmarried	109	(27.6)	75	(30.6)	34	(22.7)	**0.008**
Married	261	(66.1)	161	(65.7)	100	(66.7)	
Divorced/widowed	25	(6.3)	9	(3.7)	16	(10.7)	
**Children (*****n*****)**							
0	113	(28.6)	78	(31.8)	35	(23.3)	**0.006**
1–2	167	(42.3)	108	(44.1)	59	(39.3)	
≥3	115	(29.1)	59	(24.1)	56	(37.3)	
**LIFESTYLE AND HEALTH FACTORS**							
**Smoking habit**							
Yes	140	(35.4)	101	(41.2)	39	(26.0)	**0.006**
No	247	(62.5)	139	(56.7)	108	(72.0)	
Ex	8	(2.0)	5	(2.0)	3	(2.0)	
**Comorbidities (including obesity)**							
0	278	(70.4)	170	(69.4)	108	(72.0)	0.581
≥1	117	(29.6)	75	(30.6)	42	(28.0)	
**BMI**							
<18.5	11	(2.8)	4	(1.6)	7	(4.7)	**0.030**
18.5–24.99	173	(43.8)	100	(40.8)	73	(48.7)	
25–29.99	130	(32.9)	92	(37.6)	38	(25.3)	
≥30	81	(20.5)	49	(20.0)	32	(21.3)	
**Blood pressure**							
Systolic ≥ 140 mmHg	62	(15.7)	48	(19.6)	14	(9.3)	**0.007**
Mean systolic (mmHg)	121		125		114		**<0.001**
Diastolic ≥90	70	(17.7)	53	(21.6)	17	(11.3)	**0.009**
Mean diastolic (mmHg)	76		79		72		**<0.001**
**QTc**							
Mean (ms)	378.11		372.02		388.07		**< 0.001**
Normal	382	(96.7)	235	(95.9)	147	(98.0)	0.386
Borderline/Long	13	(3.3)	10	(4.1)	3	(2.0)	
**Blood glucose**							
Mean (mg/dL)	90.1		93.0		85.4		**0.007**
≥100 mg/dL	53	(13.4)	41	(16.7)	12	(8.0)	**0.010**

*Bold values indicate significant results*.

Regarding the educational level, the majority of the sample, 240 (60.8%) individuals (159 men and 81 women) had middle school.

Over 395 subjects, 109 (75 men and 34 women) were unmarried, 261 (161 men and 100 women) were married, 25 (9 men and 16 women) were divorced or widowed. Concerning the number of children, 113 subjects (78 men and 35 women) had no children, 167 (108 men and 59 women) had 1 or 2 children, 115 (59 men and 56 women) had more than 2 children.

Regarding lifestyle habits, it has been shown that 101 men (41.2% of male subjects) and 39 women (26% of female subjects) were smokers.

After calculating the BMI, it was 26.55 ± 5.11 (mean ± SD) in men and 25.9 ± 5.74 in women with no statistically significant difference (*p* = 0.256). The majority of men (57.6%) showed as overweight/obese while most women (53.3%) were normal/underweight.

Concerning health status, 117 participants (75 men and 42 women) showed at least one chronic disease including hypertension (21 subjects, 20 men and 1 woman), diabetes (13 subjects, 12 men and 1 woman), cardiovascular diseases (7 subjects, 4 men and 3 women), respiratory diseases (5 subjects, 2 men and 3 women), cancer (4 subjects, 3 men and 1 woman), autoimmune diseases (4 subjects, 2 men and 2 women), neurological diseases (3 subjects, 2 men and 1 woman), obesity (81 subjects, 49 men and 32 women). Blood tests revealed 53 individuals (41 men and 12 women) with a glucose level ≥ 100 mg/dL.

Dealing with blood pressure 62 subjects (48 men and 14 women) had a systolic blood pressure ≥ 140 mmHg and 70 individuals (53 men and 17 women) had a diastolic blood pressure ≥90 mmHg.

About QTc intervals we found 9 subjects (6 men and 3 women) with borderline values and only 4 (all men) with a long QTc interval.

### Perceived Stress Scale

**Figure 1:**
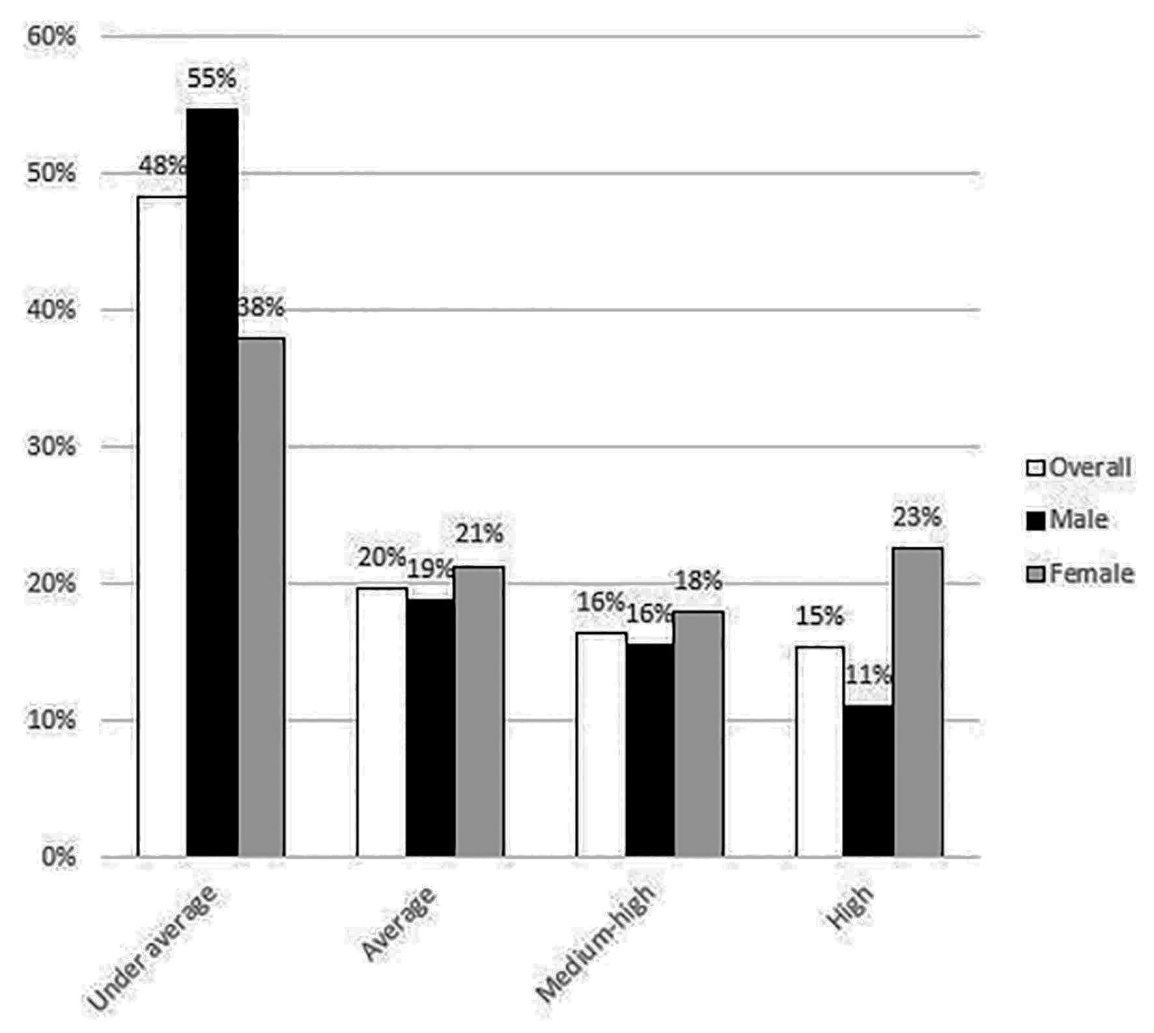
Sample distribution according to gender in the perceived stress scale categories.

As reported in Table 2, generally considering the whole sample, most of the participants (68%) showed a under average/average PSS-10 score, but this percentage highlights a gender difference, as it represents 73.5% of the men group and 59.3% of women (Figure 1). In particular, 11% men and 22.7% women showed high PSS values, therefore the percentage resulted double among women.

**Table 2:** Perceived stress scale score in a population of unemployed subjects (*N* = 395).

		**Study population**		**M**		**F**		
		***N***	**%**	***N***	**%**	***N***	**%**	***p*-value**
**Perceived stress scale**								
**PSS**	Under average	191	48.4	134	54.7	57	38.0	**0.003**
	Average	78	19.7	46	18.8	32	21.3	
	Medium-High	65	16.5	38	15.5	27	18.0	
	High	61	15.4	27	11.0	34	22.7	

*Bold values indicate significant results*.

Afterwards, taking into account PSS mean values and dividing the sample by different categories (age groups, education, marital status, number of children, smoking habit, comorbidities, and BMI), a comparison between men and women groups was performed. Results are reported in Table 3.

**Table 3:** Mean difference in perceived stress between genders, according to sociodemographic, lifestyle, and health factors.

	**M**			**F**				
	***N***	**Mean PSS**	**SD**	***N***	**Mean PSS**	**SD**	***p*-value**	**CI 95%**
**Total**	245	10.71	6.1	150	13.11	6.5	**<0.001**	1.17–3.73
**Age groups**								
19–39	116	10.50	5.80	91	13.30	6.37	**0.002**	1.24–4.47
40–59	100	10.82	6.41	56	12.71	6.39	0.069	−0.22–4.01
≥60	29	11.14	6.80	3	17.33	10.02	0.182	−2.55–14.94
**Education**								
Illiterate	4	9.50	7.00	3	17.67	4.16	0.060	−3.67–20.00
Elementary school	16	11.81	6.92	12	14.25	6.94	0.296	−3.00–7.88
Middle school	159	10.93	6.26	81	13.65	6.34	**0.002**	1.03–4.42
High school	64	9.97	5.79	49	11.71	6.77	0.152	−0.60–4.09
University	2	10.00	1.41	5	12.40	5.86	0.690	−8.95–13.75
**Marital status**								
Unmarried	75	11.12	5.72	34	13.53	7.13	0.158	−0.13–4.95
Married	161	10.61	6.38	100	12.83	6.54	**0.004**	0.61–3.84
Divorced/widowed	9	9.00	5.79	16	13.94	4.97	**0.030**	0.40–9.48
**Children (*****n*****)**								
0	78	11.38	5.58	35	14.23	6.84	0.067	0.43–5.26
1–2	108	10.69	6.79	59	11.98	6.64	0.170	−0.86–3.45
≥3	59	9.85	5.63	56	13.59	6.08	**<0.001**	1.58–5.91
**Smoking**								
Yes	101	10.93	6.85	39	14.03	6.06	**0.005**	0.62–5.57
No	139	10.58	5.70	108	12.77	6.61	**0.012**	0.64–3.73
Ex	5	9.60	3.29	3	13.33	10.02	0.416	−7.66–15.13
**Comorbidities (including obesity)**								
0	170	10.52	6.17	108	13.73	6.58	**<0.001**	0.99–3.71
≥1	75	11.13	6.14	42	11.50	6.11	>0.999	−0.21–8.26
**BMI**								
<18.5	4	13.00	6.98	7	12.86	6.74	0.927	−9.82–9.53
18.5–24.99	100	10.44	5.54	73	12.89	6.13	**0.013**	0.69–4.21
25–29.99	92	10.35	6.72	38	15.55	6.88	**<0.001**	2.62–7.88
≥30	49	11.73	6.23	32	10.75	6.15	0.515	−3.79–1.82
**Blood pressure**								
Systolic ≥140 mmHg	48	10.67	6.54	14	13.57	7.11	0.095	−1.15–6.96
Systolic <140 mmHg	197	10.72	6.08	136	13.06	6.47	**<0.001**	0.97–3.71
Diastolic ≥90	53	10.81	6.18	17	13.29	7.19	0.161	−1.10–6.06
Diastolic <90	192	10.68	6.16	133	13.08	6.45	**<0.001**	1.01–3.80
**QTc**								
Normal	235	10.64	6.18	147	13.10	6.57	**<0.001**	1.15–3.77
Borderline/Long	10	12.30	5.50	3	13.67	2.08	>0.999	−5.95–8.69
**Glucose**								
≥100 mg/dL	41	10.59	6.43	12	13.83	5.87	0.126	−0.91–7.41
<100 mg/dL	204	10.73	6.12	138	13.04	6.58	**<0.001**	0.95–3.68

*Bold values indicate significant results*.

As a result, we divided the sample by gender and, considering PSS score mean values, a comparison between subgroups inside the same sociodemographic, lifestyle, and health factors categories was performed. The only statistically significant difference was found in the women group between overweight and obese female individuals (*p*-value 0.003).

Individual logistic regression models examining the association between each population characteristics and the development of moderate/severe perceived stress were constructed. This analysis showed that among the numerous characteristics analyzed, only gender was associated with higher perceived stress levels in this population (Table 4).

**Table 4:** Crude OR using univariate logistic regression.

	**Moderate/severe perceived stress**		
**Variable**	**OR (95% CI)**	***p*-value**	**C**
**Gender**			0.577
M	1.0	Ref.	
F	1.898 (1.232–2.932)	**0.004**	

*Bold values indicate significant results*.

## Discussion

The present study evaluated perceived stress along with health status and associated factors with a gender perspective in a sample of 395 southern Italian subjects nearly engaged for a job after a period of unemployment.

Most participants (68%) showed with a under average/average PSS-10 score, meaning that our data are overlapping with PSS-10 values in the developed countries general population (11–13). Considering the existing literature, the results are not in line with the starting hypothesis. Indeed, high levels of perceived stress were expected in unemployed subjects because the lack of employment in those who desire to work has been reported to be associated with increased perceived stress. Taking into account these considerations, it has to be remarked the fact that the study population has been recruited in occasion of a mandatory preventive medical check prior to employment finalization, and would have started working in the short term; this expectation may have contributed to buffer the perceived stress level that was expected in an unemployed population.

Mean PSS-10 values in our study were 10.71 in men and 13.11 in women with a statistically significant difference (*p* < 0.001). The percentage of women showing high PSS values resulted double than men, as confirmed by the association between female gender and higher levels of perceived stress indicated by the application of logistic regression models. This result parallels other studies showing that women report more stressful life events (20). Large population-based European studies have also found higher mean PSS scores among women compared with men (12–14).

Stress appears to be differently experienced between genders: emotional exhaustion prevails in women, while men tend to feel more depersonalized. Actually, women seem to be in greater risk for psychological problems, due to the combination of biological and social determinants; these include gender stereotypes, inequity, social segregation, and autonomy (31).

A European multicentric study highlighted gender and regional differences in perceived stress, explaining them with an interaction among culture, economic environment and work organization (32).

In the present study, mean PSS in male subjects showed an increasing trend with age; whereas in women it suggested a decrease with age, though only two of the three women over 60 years presented high PSS values. The three national-level surveys in a United States (US) study (11) reported an increase in the PSS scores with decreasing age, explained by authors as we grow older, we both interpret events as less stressful and develop better coping strategies. Also the study based on a representative survey of the German population (13) highlighted that perceived stress was highest among younger. Whilst these reports are similar to those we found in female subjects; in the male group of our study the perceived stress tends to increase with age. A possible explanation is that the frustration of not being able to find a job grows over time, more in male than in female individuals.

According to the ultimate available OECD data in Italy, higher education level parallels to lower unemployment rate, following the same trend of the European Union countries (33). In this study, only 7 subjects were illiterate and 7 were graduated. Excluding these two extremes, it is possible to observe a decreasing mean PSS with the increasing of the education level, both in men and women. These findings are widely in accordance with other studies in which psychological stress increased in a graded fashion with decreasing education (11) and perceived stress is highest among less educated participants (13). However, as mentioned above, the low perceived stress found in most subjects of our population does not allow to suggest an association with unemployment.

Previous results (13, 34) underlining a high perceived stress in unmarried and divorced subjects are not consistent with our findings, which did not highlight a role of marital status.

Literature reports (13, 14), though not relating perceived stress with children number, are contradictory in considering children a stressor. In this study, participants with children did not feel more stressed; conversely, higher stress was observed in childless subjects, particularly women. This finding may be explained by the controversial psychosocial hypothesis of motherly feel.

A cross-sectional, community-based study based on data from the World Health Survey suggested that perceived stress is significantly associated with higher smoking rates in Africa, Americas, and Asia, but not in Europe. However, in an analysis performed in a representative German sample, unemployment was associated with a 46% higher probability to smoke (35). Our results do not suggest an association between perceived stress and smoking habit in men, whereas stress perception was higher in smoking women.

According to numerous studies and several systematic reviews and metanalyses demonstrating a negative association between unemployment and health (3, 6, 36–38), we found higher stress in subjects who suffered from at least one chronic disease; no statistically significant difference was found intra- and inter-genders.

Stubbs et al. (39) analyzed data from the World Health Organization Study on Global Aging and Adult Health (SAGE), a survey aiming to investigate the relationship between perceived stress, chronic conditions and multimorbidity (i.e., ≥2 chronic conditions) in low and middle income countries. In that sample aged ≥50, authors identified that over a half had multimorbidity, and greater numbers of chronic conditions were associated with higher levels of stress in a dose-dependent mode. However, hypertension and obesity were not significantly associated with stress in any of the countries. As regards the effect of body weight on perceived stress, literature is not conclusive. In a nationally representative sample of young adults in USA (40), it has been observed that perceived stress is inversely associated with BMI and waist circumference only among men, while others underlined a significant positive relation for both genders (41) or positive associations only for women (42). In our study, the percentage of overweight or obese subjects was differently represented in the two genders (57.6% among men and 46.7% among women); proceeding with the comparison between BMI subgroups, we found stress over average only in overweight women, but unexpectedly not in obese subjects. The reciprocal relation between eating and stress is very complex; it is known that food can become a coping strategy for many individuals, which may explain low stress levels in this population.

AS stress is a potential risk factor also for cardiovascular and neuroendocrine system, these targets were investigated by performing ECG, blood pressure, and glucose measurement.

A study (30) exploring QT interval parameters suggested that conditions associated with work-related stress can have subclinical effects on the autonomic regulation of cardiac function. We found only a few male individuals with long QTc intervals, so an inter-gender comparison is not applicable; despite we found only 6 men and 3 women with borderline QTc values, we can highlight that males showed a higher mean PSS than those with normal QTc. However, due to low stress level in this population, a significant autonomic imbalance was not observed.

Though the relation between stress and hypertension has been widely investigated, it is still a matter of debate (43). No intra-gender variability in PSS scores in all subjects for either systolic or diastolic blood pressure was reported in this population.

Though preliminary results (44) indicated that perceived work-related stress could be associated with increased blood glucose levels, in our results blood glucose was not associated to perceived stress level.

This is, to our knowledge, the first study investigating the relation between stress and unemployment in a middle-aged population of southern Italy with a gender perspective, as other reports (11–13) associated unemployment as a dependent variable to perceived stress.

There are few published surveys focusing on potential geographical and gender differences within specific stressors. They all concluded confirming differences in stress perception according to gender and regional factor; however, both these variables appeared to be weak independent predictors of perceived stress at work (32).

Nonetheless, focusing the complex facets of stress (cultural factors affecting stress perception through coping strategies, home/work interface, social support vs. stigma toward the unemployed subjects, as well as economic environment) is not straightforward; a quantitative instrument administered with standard modalities is presumably inadequate to this purpose. For this reason, we recognize certain limitations to our study. It is a non-prospective observational study, so temporal relationship between perceived stress and the various stressor variables cannot be determined. Also, we did not have the possibility to evaluate PSS over time in order to assess how working activities might have changed perceived stress in an unemployed population. Moreover, the study did not consider social economic status, which can influence perceived stress levels among individuals in a community, assuming that unemployed subjects had a low income and social status. But more importantly, this study design and the questionnaire administered did not allow to focus on other important social aspects as mentioned above.

## Conclusions

Our results highlight that females experience higher perceived stress, pointing out the need for further research investigating gender-specific components of work-related stress. This study design started from the awareness that people react and handle stress differently due to multiple individual and contextual factors which can be difficult to capture in observational studies. PSS-10 showed itself as a reliable, validated and quick scale to measure stress in population samples. However, it does not include items assessing social determinants, so stress level might be underestimated in the current study.

## Data Availability Statement

The raw data supporting the conclusions of this article will be made available by the authors, without undue reservation.

## Ethics Statement

Ethical review and approval was not required for the study on human participants in accordance with the local legislation and institutional requirements. The patients/participants provided their written informed consent to participate in this study.

## Author Contributions

CF and CC: conceptualization and methodology. SI and SM: data analysis and validation. MT, GB, and FV: investigation. GB and MP: resources. SI and AC: writing—original draft preparation. CF, CC, and SI: writing—review and editing. CF: supervision and project administration. All authors have read and agreed to the published version of the manuscript.

## Conflict of Interest

The authors declare that the research was conducted in the absence of any commercial or financial relationships that could be construed as a potential conflict of interest.
